# Evaluation of static and dynamic Pupillometry changes in men using Silodosin for benign prostatic hypertrophy

**DOI:** 10.1186/s12886-021-01894-7

**Published:** 2021-03-08

**Authors:** Umut Karaca, Engin Kaya, Onder Ayyildiz, Gokhan Ozge, Murat Kucukevcilioglu, Gulsah Usta, Fatih Mehmet Mutlu

**Affiliations:** 1 Department of Ophthalmology, Suleyman Demirel University Faculty of Medicine, Isparta, Turkey; 2 Department of Urology, University of Health Sciences, Gulhane Education and Research Hospital, Ankara, Turkey; 3 Department of Ophthalmology, University of Health Sciences, Gulhane Education and Research Hospital, Ankara, Turkey

**Keywords:** Benign prostatic hypertrophia, Automated pupillometry, Silodosin, α1 adrenergic antagonists, Intraoperative floppy iris syndrome

## Abstract

**Background:**

Intraoperative floppy iris syndrome is a variant of the small pupil syndrome that has been observed during cataract surgery in some patients currently or previously treated with α1 adrenergic blockers. It is important for cataract surgeons to predict the probable complications preoperatively. Our study aims to evaluate the static and dynamic pupil characteristics of patients treated with silodosin—a selective α1 adrenergic blocker—for benign prostate hypertrophy (BPH) and to compare these values with healthy subjects using an automatic quantitative pupillometry system.

**Methods:**

A total of 74 BPH patients treated with silodosin for six months (group 1) and 30 healthy subjects (group 2) were enrolled in this prospective multidisciplinary cross-sectional study. Static and dynamic pupillometric measurements were obtained under optimized conditions, and the results were compared between the two groups.

**Results:**

Seventy-four male patients with a mean age of 63,35 ± 7,21 (46–77) years with BPH treated with silodosin and 30 normal male subjects with a mean age of 63,07 ± 4,73 (52–71) years were analyzed. There were statistically significant differences between the groups with regard to scotopic pupil diameter (PD), high photopic PD, and low photopic PD (*p* < 0.001, for each one). The patient group had statistically significant higher values of amplitude and velocity of pupil contraction and lower values of duration of pupil contraction and latency as well as duration and velocity of pupil dilation.

**Conclusion:**

The static and dynamic pupil characteristics of subjects treated with silodosin for BPH are different from those of healthy eyes. In addition, our results may have shed light on the risk for intraoperative floppy iris syndrome (IFIS) before cataract surgery; thus, surgeons can be alert and take precautions.

## Background

Silodosin is a new subtype of selective Adrenoceptor Blocker (AB) approved for Benign Prostatic Hyperplasia (BPH) and Lower Urinary Tract Symptoms (LUTS) [1, 2]. As the other α-blockers (alfuzosin, doxazosin, tamsulosin and terazosin), silodosin has some adverse effects, including asthenia, dizziness, nasal congestion, arterial (orthostatic) hypotension and intraoperative floppy iris syndrome (IFIS) [3].

Intraoperative floppy iris syndrome is a complication characterized by abnormal movements in the iris and iris prolapse during cataract surgery [4]. Although the pathophysiology and risk factors of IFIS have not been fully elucidated, the most important known risk factor is current or previous exposure to alpha-antagonists [5]. This situation reported in approximately 5% of surgeries; is predisposing to intraoperative, −posterior capsule rupture, vitreous loss, nuclear prolapse, iridodialysis- and postoperative -iris defects, increased postoperative intraocular pressure (IOP) and endophthalmitis- complications [6]. The methods that can predict the occurrence of IFIS preoperatively; can provide an opportunity to develop the most accurate approach to prevent complications.

Pupillary constriction and dilatation is related to responses of the iris to parasympathetic and sympathetic impulses, respectively [7]. Pupillary examination by observing and measuring pupil size, shape, symmetry, response to light and response to near reflex can help clinicians to diagnose many ocular and neurological disorders like IFIS [8]. IFIS is a variant of the small pupil syndrome that has been observed during cataract surgery in some patients currently or previously treated with the α1 ABs [9].

Dynamic pupillometry is an autonomic testing tool for pupillary measurements [10]. These measurements can be taken statically and dynamically in scotopic, mesopic, or photopic conditions. Recent developments in automated pupillometry devices have enabled quantitative, objective, noninvasive, and repeatable measurements of pupil diameter (PD) as well as pupillary kinetics [11]. This study aimed to evaluate the static and dynamic pupil characteristics of patients treated with silodosin for LUTS/BPH and to compare these values with healthy subjects using an automatic quantitative pupillometry system.

## Methods

The study was designed as a prospective multidisciplinary cross-sectional study and carried out from July 2015 to July 2017 at the ophthalmology and urology clinics of a tertiary hospital. The study protocol was approved by the Gulhane Education and Research Hospital Clinical Research Ethics Committee and carried out in accordance with the Declaration of Helsinki. Written informed consent was obtained from each individual participant.

A total of 74 BPH patients treated with silodosin for six months (group 1) and 30 healthy subjects (group 2) were enrolled in the study. BPH patients were examined by the Urology Department. The inclusion criteria were as follows: male patients ≥45 years with symptomatic BPH, a peak flow rate (Qmax) of < 15 ml/s, International Prostate Symptom Score (IPSS) of ≥8, quality of life score (QLS) of ≥3, and a peak flow rate (Qmax) of < 15 ml/s. Patients with severe hepatic or renal insufficiency, urinary tract infections, urethral stricture, neurogenic bladder, and a history of urethral or prostatic surgery or the use of various alpha blockers were excluded by the urologist from the study, initially.

Afterwards, all the subjects underwent a comprehensive ophthalmic examination, including the measurement of the uncorrected and best corrected visual acuity and slit-lamp biomicroscopy. Individuals with a history of systemic vasculopathies such as hypertension or diabetes mellitus, ocular inflammation, anisocoria, head/orbital trauma or ocular surgery/laser treatment were excluded. Those with iris and/or pupil abnormalities, such as coloboma, anterior–posterior synechia and pseudo exfoliation syndrome were excluded. Patients with glaucoma or taking medications that may affect iris mechanics, such as tropicamide, cyclopentolate, pilocarpine and narcotic-derived medications, were excluded. Individuals with neurological disease or other diseases of the visual pathways and those who were not cooperative enough to undergo pupillometry examinations were also excluded.

A technician blind to the study performed pupillometry measurements using the same automatic quantitative pupillometry system (MonPack One, Vision Monitor System, Metrovision, Pérenchies, France). Average values were selected after three consecutive measurements for each participant using the automatic-release mode of the device. All measurements were performed at the same time of the day (12:00 am – 01:00 pm). The following parameters were recorded: latency and duration of contraction and dilatation (ms); initial, minimum, maximum and mean pupil diameter (PD) (mm); amplitude of contraction (mm); and contraction and dilatation speed (velocity) of the pupil (mm/s). Static pupillometry measurements were obtained under several illumination levels to measure pupil size in scotopic (0.1 cd/m2), mesopic (1 cd/m2), low photopic (10 cd/m2), and high photopic (100 cd/m2) vision conditions. Scotopic PD, mesopic PD, low photopic PD and high photopic PD values were recorded. In darkness, after five minutes of darkness adaptation, dynamic pupillometry measurements were obtained for a duration of 90 s.

### Statistical analysis

Statistical Package for Social Sciences (SPSS) version 20.0 for Windows (IBM, New York, USA) was used for data analysis. The statistical significance was set at *p* < 0.05. Descriptive statistics were presented as mean ± standard deviations, frequency distributions and percentages. The normal distribution of the variables was tested using analytical methods (Kolmogorov–Smirnov/ Shapiro–Wilk tests). An independent sample t-test was used to compare quantitative data.

## Results

Seventy-four male patients with a mean age of 63,35 ± 7,21 (46–77) years with BPH treated with silodosin and 30 normal male subjects with a mean age of 63,07 ± 4,73 (52–71) years were analyzed. There were no significant differences between the groups with regard to age (*p* = 0.57).

Table 1 shows the static pupillometry measurements of Group 1 (patient) and Group 2 (control). There were statistically significant differences between the groups with regard to scotopic PD, high photopic PD, and low photopic PD (*p* < 0.001, for each one). Mesopic PD was not statistically significant despite the powerful difference. (*p* = 0.007).

**Table 1:** Static Pupillometric Results of Two Groups. (PD: Pupil Diameter, mm: millimeter, ms: milliseconds)

	Group 1 (n: 74)	Group 2 (n:30)	P
Scotopic PD (mm)	5,08 ± 0,28	5,53 ± 0,86	< 0,001
Mesopic PD (mm)	4,17 ± 0,27	4,55 ± 0,90	0,007
Low Photopic PD (mm)	3,17 ± 0,26	3,66 ± 0,57	< 0,001
High Photopic PD (mm)	2,18 ± 0,34	2,85 ± 0,46	< 0,001

Dynamic pupillometric measurements of the groups are shown in Table 2. The patient group had statistically significant higher values of amplitude and velocity of pupil contraction and lower values of duration of pupil contraction and latency, duration and velocity of pupil dilation. On the other hand, resting diameter values were not statistically significant, but there was a powerful difference between groups (*p* = 0.007). There were no significant differences between the groups with regard to latency of pupil contraction (*p* = 0.895).

**Table 2:** Dynamic Pupillometric Results of Two Groups. (mm: millimeter, ms: milliseconds)

	group 1 (n:74)	Group 2 (n:30)	p
Resting Diameter (mm)	4,11 ± 0,22	4,33 ± 0,57	0,007
Amplitude of Pupil Contraction (mm)	1,70 ± 0,32	1,41 ± 0,43	< 0,001
Latency of Pupil Contraction (ms)	284,2 ± 22,1	281,8 ± 97,6	0,895
Duration of Pupil Contraction (ms)	532,3 ± 49,5	677,1 ± 186,2	< 0,001
Velocity of Pupil Contraction (ms)	5,03 ± 0,37	4,34 ± 1,18	0,002
Latency of Pupil Dilation (ms)	851,1 ± 101,5	972,2 ± 183,0	< 0,001
Duration of Pupil Dilation (ms)	1603,3 ± 73,5	2921,9 ± 112,7	< 0,001
Velocity of Pupil Dilation (ms)	1,70 ± 0,09	2,42 ± 0,95	< 0,001

## Discussion

In this study, we used an automatic system for static and dynamic pupillometry measurements on the cases with BPH treated with silodosin—a selective α-blocker—and healthy subjects to determine the differences in static and dynamic pupil characteristics. To the best of our knowledge, this is the first study to evaluate the static and dynamic pupil characteristics in subjects treated with silodosin using an automatic quantitative pupillometry system (Vision Monitor System, Metrovision).

Pupillary examination, including pupil size, shape, symmetry, response to light, and response to near reflex, is important before planning intraocular surgery. However, the subjective examination of pupils can be affected by several factors, such as illumination and the examiner’s experience [12]. Pupillary response to light can be measured by using an automatic pupillometry system and controlling lightening conditions, and multiple, quantitative measurements can be obtained. This improves the repeatability of the measurements, solves the problem of examiner-dependent errors and reduces false negative responses [13, 14].

IFIS is a variant of the small pupil syndrome and was first described by Chang et al. in 2005 [9]. Previous studies have shown that tamsulosin and other ABs inhibit phenylephrine-induced mydriasis, causing myosis to almost equal extents and duration, regardless of dose [15]. An important mechanism of IFIS is drug– melanin interaction causing dilator muscle atrophy [16]. Silodosin is a novel, more selective alpha-blocker, which is specific to the lower urinary tract and may have fewer side effects than the other alpha-blockers [17, 18]. Despite this selectivity, Ipekci and Chatterjee reported silodosin-associated IFIS in their cases similar to other AB’s [19, 20]. A clinically poor dilated and floppy iris during surgery may shrink the visualization of the surgical field and complicate the surgery [21]. This clinically observed pupillary changes had not ever been observed with quantitative pupillometric analysis before.

The following parameters were measured with an automatic pupillometry system: pupil diameter before and after light stimulus; latency, duration, velocity and amplitude of pupillary constriction; and velocity, latency and duration of pupillary dilatation. Amplitude and maximum constriction velocity reflect the active parasympathetic part of the light reflex, whereas the dilatation velocity reflects the active sympathetic part [7].

In this study, all static PDs, including the scotopic, mesopic low and high photopic PDs, were smaller in the patient group. Furthermore, the present study found that patients had higher resting PDs than the healthy groups. Since the pupillary resting diameter reflects the balance between sympathetic and parasympathetic autonomic systems, it can be said as a result of this study that silodosin disrupts the balance between autonomic systems in the direction of the parasympathetic system. Dogan et al. investigated the effects of tamsulosin hydrochloride and alfuzosin on pupil diameters and reported smaller resting pupil diameter sizes with alfuzosin treatment especially [22].

This study investigated pupil dynamics, including latency, duration, and velocity of pupil constriction and redilation of patients treated with silodosin. Our results indicated that patients showed statistically significantly higher differences with regard to amplitude of pupil contraction and velocity of pupil contraction. Duration of pupil contraction, as well as values of duration, velocity and latency of pupil dilatation, was statistically significantly lower when compared with healthy eyes. Pupillary dynamics (amplitude and velocity of constriction and dilatation) are a function of the balance between sympathetic and parasympathetic tone in which increased sympathetic balance decreases the constriction velocity, whereas increased parasympathetic balance increases it [23]. These results may suggest that silodosin alters the pupillary kinetics and could be responsible for IFIS as a side effect.

This study had a number of limitations. The relatively small number of patients in the control group could affect the validity and importance of the comparisons. The fact that the pupillometry system used in the study requires full compliance of the patients may affect the results. It is important to have an experienced technician so that this situation does not affect the work. Another disadvantage of the study is that PD differences, such as physiological anisocoria, can be seen even in completely healthy subjects.

In conclusion, this study revealed that static and dynamic pupil characteristics of subjects treated with silodosin for BPH differ from those of healthy eyes. In addition, our results may have shed light on the risk of IFIS before cataract surgery; thus, surgeons can be alert and take precautions. There have been limited studies comparing the effects of silodosin in the literature, so further long-term studies are required to clarify the effects of silodosin on static and dynamic pupillary functions.

## Data Availability

The datasets used and/or analyzed during the current study available from the corresponding author on reasonable request. Unfortunately, the data is not publicly available due to local data protection laws.
